# The pivot: Fighting Irish fighting COVID-19

**DOI:** 10.1371/journal.ppat.1009390

**Published:** 2021-03-10

**Authors:** Kasturi Haldar

**Affiliations:** University of Notre Dame, Notre Dame, Indiana, United States of America; University of Colorado Denver, UNITED STATES

“Doing something difficult and reaching our goal brought us closer and made us stronger. I hope people look back and go, wow, look at what we did!”—Dr. Marie Lynn Miranda, Charles and Jill Fischer Provost, University of Notre Dame, Director Children’s Environmental Health Initiative.

The Coronavirus Disease 2019 (COVID-19) pandemic caused by the emergence of the Severe Acute Respiratory Syndrome Coronavirus 2 (SARS-CoV-2) has presented significant challenges for university education. Provost Marie Lynn Miranda kindly agreed to an interview to share her viewpoint/opinion on the reopening of the University of Notre Dame for in-person instruction and the critical pivot that quelled an ominous infection surge, enabling all (approximately 12,600) undergraduate and graduate students to continue campus life till the conclusion of the Fall 2020 semester.

**Q1**: When did you realize that the pandemic would be a major challenge of your first year, and how did your background help you prepare for the job?

**A1**: I understood when I accepted the position in March 2020, helped by my background in public health and leading the emergency response to Hurricane Harvey as Provost at Rice University. Having been a Provost, I also understood the day-to-day responsibilities of the office, and this enabled completing a lot of important non-COVID-19 work in the fall term (although this may not have been apparent to many). That I’m statistician was very helpful to understanding every number floating around and their associated uncertainties. I could read rapidly emerging (and often changing) COVID-19 literature, become versed in the science of transmission pathways, diverse outcomes of infection, and how to apply them to students safely returning to and resuming campus life.

**Q2**: What had to be accomplished as Provost-elect (March 12 to June 30) and subsequently as Provost (July 1 to August 10) before the start of the Fall term: What were the challenges and strengths of your circumstances?

**A2**: The President, Father John Jenkins, laid out a plan that the semester would run uninterrupted from August 10 to November 20, which I fully supported. This was no doubt ambitious, and some voiced need for more consultation, but my view was that we would continuously evaluate whether we could meet the goal as we moved along. I did have to get to know a large number of people very quickly. Relationships that would have collegially matured over 12 to 18 months were instead forged in a compressed timeline. But the outgoing Provost Tom Burish provided tremendous support: We were effectively co-Provosts during the transition, and he remains an invaluable source of advice. The decisions made by Father John Jenkins, Shannon Cullinan (Executive Vice President), and myself prioritized the health and safety of all members of the Notre Dame community.

**Q3**: How did we arrive upon the first “on-campus” testing strategy that emphasized testing of symptomatic individuals in conjunction with contact tracing but a secondary role for surveillance screening (or asymptomatic testing)?

**A3**: At the time when we started, providing symptomatic testing with quarantine and isolation (QI) was recommended. It’s now recognized that asymptomatic testing is a good idea, and we’ve subsequently expanded to scale. We built a strong relationship with the local Deputy Health Director, as well as partnerships with Cleveland Clinic and Rush University Hospital. It took a while to build our surveillance/asymptomatic screening capacity. If we did it over again, we’d build surveillance/asymptomatic testing earlier.

Note: Fox and colleagues [1] provide an in-depth report of tests utilized and rationale for their deployment. Briefly, just prior to the semester’s start, nasal swab kits were mailed to students at their homes and subsequently assessed by reverse transcription polymerase chain reaction (RT-PCR) at a national laboratory. On-campus RT-PCR testing of symptomatic persons and screening of student athletes were in place by the semester’s start. The pivot, to reduce barriers to testing, led to introduction of the rapid antigen test (Sofia SARS Antigen Fluorescent Immunoassay; Quidel, California, United States of America) administered to symptomatic or “exposed” individuals. Persons negative were followed up for later nasal swabs tested by RT-PCR. The pivot also spurred nasal- and saliva-based RT-PCR surveillance of asymptomatic persons. Moreover, 86% were surveillance tests, highlighting the importance of asymptomatic screening. Scaled, saliva-based RT-PCR surveillance tests were assessed at the ND Genomics and Bioinformatics Core Facility (led by Director Prof. Michael Pfrender and Assistant Director Melissa Stephens). All tests were paid for by the University of Notre Dame.

**Q4**: Two weeks after the start of semester, infections surged (largely due to off-campus student gatherings), and on August 18, we shut down in-person and moved to online classes. At this point, you led a pivot, and in another 2 weeks, infections were controlled, and we were back to in-person classes. Tell us about the pivot.

**A4**: Well yes, we did move to online classes, but the irony of it was that the safest place on campus in terms of COVID-19 transmission was in the classroom. But going online gave us added time to focus on the expansion of our infrastructure for testing, QI, and implement a better data system to get control of the wave. There was underused hotel space because there have been no football visitors and no conferences, which allowed us to expand QI space as we needed it. Notre Dame provided the cleaning staff as well as personnel for monitoring and supporting students (e.g., meal services) through QI.

We also needed to communicate with the students that as a community we had to come back and that they were our partners in this. Communication with students was done at all levels. The President, Father Jenkins, and different people in Student Affairs spoke to them. I did a video with Father Pete, explaining the science and what we wanted from them and how/why they were are our partners. We provided faculty with slides to incorporate into class instruction every week to kindle discussion regarding hygiene, the essentiality of masks and social distancing, and the importance of being accountable to our communities. Even emergency weather alerts incorporated COVID-19 consistent guidelines.

University of North Carolina (Chapel Hill) and North Carolina State had a surge in infections around the same time as we did. They sent their students home. We didn’t want to do that. We felt it was the right thing to try to correct the situation on campus. In fact, shortly after, Dr. Fauci said it would not be a good idea to send infected students back to their home communities.

**Q5**: How disruptive was football and student exit testing at the end of the semester? Did these and/or other activities lead to subsequent increased infection rates on campus (but these were well below the first wave)?

**Fig 1:**
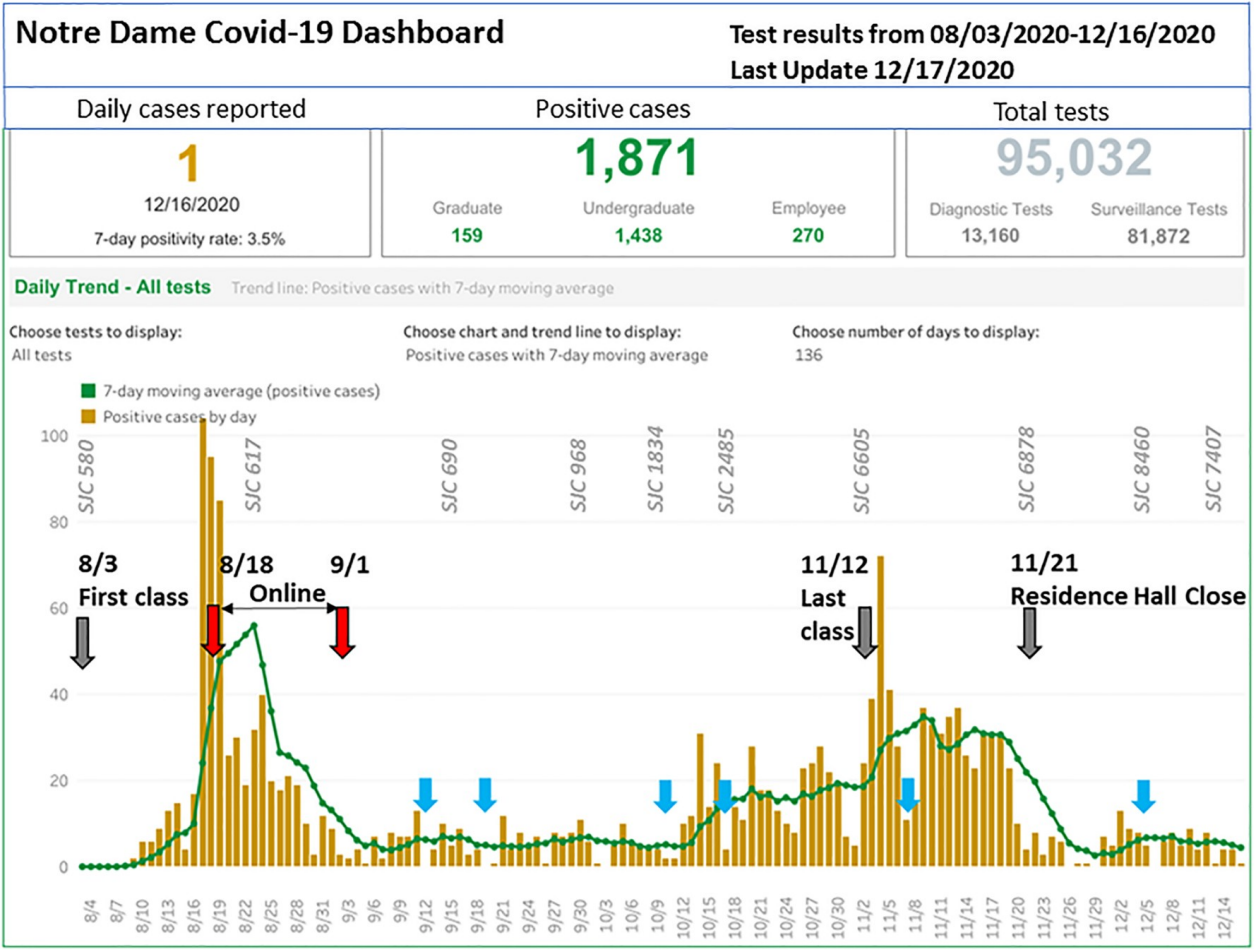
ND COVID-19 Dashboard Fall 2020 (https://here.nd.edu/our-approach/dashboard/fall-2020-data/) with data for all (diagnostic and surveillance) on-campus COVID-19 tests posted as of December 16, 2020. Top panel presents snapshots of the daily case, total positive cases, and total number of tests administered. Lower panel shows daily cases (brown histogram) and 7-day averages (green line) from August 3 to December 15, 2020. A subset of daily cases for SJC (https://www.nytimes.com/interactive/2020/us/indiana-coronavirus-cases.html) where ND is located are indicated in gray, italicized text. Dark gray arrows and associated dates respectively indicate timing of ND’s first and last classes and closing of undergraduate residence halls (marking undergraduate student departure from campus). Blue arrows indicate dates of football games (where attendance required masks and social distancing). COVID-19, Coronavirus Disease 2019; ND, Notre Dame; SJC, St. Joseph County. The data reveal an increase in infections detected through on-campus testing with sharp upward trajectory within 2 weeks after the onset of classes (August 3 to 18; but without significant increase in daily cases for St. Joseph County [SJC]) which led to shutting down in-person and moving to online instruction, as shown. The infection surge was successfully controlled, and in-person classes resumed on September 2, 2020. Football games (blue arrows) were not found to associate with increased infections. Infection increases seen mid-October through November 21, 2020 (but at lesser levels seen during the August surge) occurred with concomitant increases in COVID-19 in SJC through this period. The departure of undergraduate students led to rapid decline in campus infection levels (but it should be noted that this also greatly reduced the number of persons on campus). Over the course of approximately 12 weeks, of 1,871 positive cases, approximately 78%, approximately 8.5%, and approximately 14.4% were respectively ascribed to undergraduates, graduate students, and other employees. These findings were based on 95,032 tests where approximately 86% were surveillance and approximately 14% were diagnostic tests, emphasizing the importance of also monitoring asymptomatic infections.

**A5**: If you look at the data on website (https://here.nd.edu/our-approach/dashboard/fall-2020-data/) (also see Fig 1), the August peak came down and stayed down, including after football games. There were extensive guidelines on safe practices in the stadium. The issue was not so much when students were in stadium, but what they were doing before and after. In anticipation, a lot of people worked on 3 days on either side of the game to capture aspects of pre- and postgame festivities. The other issue is that infection rates have gone up in St. Joe County. As I’ve explained to students, there is increased risk with every trip you take to grocery store or drug store. I know there are other reasons why infections have increased on campus. Some students are tired of wearing masks all the time: Their gatherings now are small with their best buddies.

The exit plan was very well executed by a team led by 2 outstanding staff to help students comply with requirements of the states and/or countries they were returning to. It also enabled exit testing for faculty who want to participate.

**Q6**: What were unexpected benefits/opportunities, how can what we learned benefit other universities?

**A6**: COVID-19 made us innovate and change and work across unit lines much more than usual. It made us much more open to try new ways if something didn’t work. It really created an innovator mentality—not innovating for no reasons, but willing to continuously try in the face of new challenges. It brought us all (students, postdoctoral fellows, faculty, and staff) closer together. We were counting down to and exhaled on November 20 (last day for undergraduate student departure). Doing something difficult and reaching our goal brought us closer and made us stronger. I hope people look back and go, wow, look at what we did!

Others can learn that an infection surge can be controlled. A manuscript with the data has been submitted and is presently under review at the Centers for Disease Control (CDC). COVID-19 has provided opportunities for institutions to show their commitment to higher education and the research mission. I feel so proud of our faculty, graduate students, postdoctoral fellows, and research staff for their dedication to research and to all the students and faculty for staying fully in and engaged with the educational mission.
